# Identification, Mapping, and Genetic Diversity of Novel Conserved Cross-Species Epitopes of RhopH2 in *Plasmodium knowlesi* With *Plasmodium vivax*

**DOI:** 10.3389/fcimb.2021.810398

**Published:** 2022-01-13

**Authors:** Md Atique Ahmed, Gauspasha Yusuf Deshmukh, Rehan Haider Zaidi, Ahmed Saif, Mohammed Abdulrahman Alshahrani, Syeda Wasfeea Wazid, Saurav Jyoti Patgiri, Fu-Shi Quan

**Affiliations:** ^1^ Indian Council of Medical Research (ICMR)-Regional Medical Research Centre, North East Region (NER), Dibrugarh, India; ^2^ Department of Biotechnology and Microbiology, National College, Tiruchirapalli, India; ^3^ Department of Clinical Laboratory Sciences, Faculty of Applied Medical Sciences, Najran University, Narjan, Saudi Arabia; ^4^ Arogya Society of Health, Welfare and Support (ASHWAS), Assam, India; ^5^ Department of Medical Zoology, School of Medicine, Kyung Hee University, Seoul, South Korea; ^6^ Medical Research Center for Bioreaction to Reactive Oxygen Species and Biomedical Science Institute, School of Medicine, Graduate School, Kyung Hee University, Seoul, South Korea

**Keywords:** *Plasmodium knowlesi*, *Plasmodium vivax*, conserved cross-species, polymorphism, rhopH2, vaccine

## Abstract

Malaria is a major public health concern, and any tangible intervention during the pre-elimination phase can result in a significant reduction in infection rates. Recent studies have reported that antigens producing cross-protective immunity can play an important role as vaccines and halt malaria transmission in different endemic regions. In this study, we studied the genetic diversity, natural selection, and discovered novel conserved epitopes of a high molecular weight rhoptry protein 2 (RhopH2) in clinical samples of *Plasmodium knowlesi* and *Plasmodium vivax* cross-protective domains, which has been proven to produce cross-protective immunity in both species. We found low levels of nucleotide diversity (*P. knowlesi*; π ~ 0.0093, SNPs = 49 and *P. vivax* π ~ 0.0014, SNPs = 23) in *P. knowlesi* (n = 40) and *P. vivax* (n = 65) samples in the *PkRhopH2* cross-protective domain. Strong purifying selection was observed for both species (*P.* knowlesi; dS - dN = 2.41, p < 0.009, *P. vivax*; dS - dN = 1.58, p < 0.050). *In silico* epitope prediction in *P. knowlesi* identified 10 potential epitopes, of which 7 epitopes were 100% conserved within clinical samples. Of these epitopes, an epitope with 10 amino acids (QNSKHFKKEK) was found to be fully conserved within all *P. knowlesi* and *P. vivax* clinical samples and 80%–90% conservation within simian malaria ortholog species, i.e., *P. coatneyi* and *P. cynomolgi*. Phylogenetic analysis of the PkRhopH2 cross-protective domain showed geographical clustering, and three subpopulations of *P. knowlesi* were identified of which two subpopulations originated from Sarawak, Malaysian Borneo, and one comprised only the laboratory lines from Peninsular Malaysia. This study suggests that RhopH2 could be an excellent target for cross-protective vaccine development with potential for outwitting strain as well as species-specific immunity. However, more detailed studies on genetic diversity using more clinical samples from both species as well as the functional role of antibodies specific to the novel conserved epitope identified in this study can be explored for protection against infection.

## Introduction

Malaria is a vector-borne disease which is prevalent in more than a hundred countries with 228 million malaria cases and an incidence of 405,000 deaths in 2019, the majority of which are due to *Plasmodium falciparum* infections (World Health Organisation, 2019). Controlling the spread of malarial infection can result in changes in species distribution patterns; for example, in one area, the spread of *P. falciparum* and *P. vivax* has decreased dramatically, but the spread of the zoonotic malaria, *P. knowlesi*, has increased significantly in Southeast Asian countries (Ahmed and Cox-Singh, 2015; Yusof et al., 2016). *P. knowlesi*, for example, caused 5% of malaria cases in Sabah in 2004, but 98% in 2017 (William et al., 2013; Cooper et al., 2020). This simian malaria parasite, *P. knowlesi*, was reported as a major cause of human malaria in Sarawak, Malaysia, in a paper published 16 years ago (Singh et al., 2004). Since then, several Southeast Asian countries have reported zoonotic malaria cases due to *P. knowlesi*. The whole-genome and genetic studies on *P. knowlesi* identified that there are at least three subpopulations in clinical samples from Malaysia, and out of these, two of them are linked to the primary monkey hosts, *Macaca nemestrina* and *Macaca fascicularis* (Ahmed et al., 2014; Assefa et al., 2015; Pinheiro et al., 2015; Ahmed et al., 2016)

The first clinical manifestation due to malaria starts during the asexual stages of the parasite when merozoites are released from RBCs. The invasion of the parasite into red blood cells (RBC) is a complex process which engages proteins on the merozoite surface and sequentially releases them from the apical organelles (micronemes and rhoptries) (Quintana et al., 2018). During merozoite egress and host cell invasion, invasive malaria merozoites have a typical apical complex set of secretory organelles that are discharged in a tightly controlled and highly regulated order (Sherling et al., 2019). Among the prominent organelles, the rhoptries are club-shaped, twinned structures which have a bulbous body that narrows to a narrow neck as it approaches the merozoite’s apical prominence (Sherling et al., 2019). Rhoptry proteins are essential for the *Plasmodium* parasite’s ability to enter and replicate in human red blood cells (RBCs). These proteins are also involved in the invasion of target cells by sporozoites, such as mosquito salivary glands and mammalian hepatocytes (Ishino et al., 2019). PkRhopH2, a high molecular mass protein in the rhoptries (161 kDa), was found to be highly immunogenic (with cross-protective immunity) with growth inhibitory activities (Muh et al., 2020). Host cell attachment and tight-junction formation are mediated by rhoptry neck proteins; however, the function of rhoptry bulb proteins is unclear due to a lack of functional studies (Ghosh et al., 2017). More than 30 rhoptry proteins have been identified in *P. falciparum* to date (Counihan et al., 2013). RhopH2 localizes to the bulb region and interacts with RhopH3, RhopH1, the erythrocyte cytoskeleton, and exported proteins that are involved in the remodeling of the host cell leading to increase in permeability in RBCs (Counihan et al., 2013).

*P. knowlesi* and *P. vivax* have a close phylogenetic relationship with 89% gene orthologs between them (Tachibana et al., 2012). Thus, these ortholog genes with roles in red blood cell invasion are proposed as attractive cross-species vaccine candidates (Cornejo and Escalante, 2006; Carlton et al., 2008). The cross-reactivity between *P. falciparum* and *P. vivax* is due to the presence of common or similar shared B and T-cell epitopes and homology between the plasmodial proteins (Diggs and Sadun, 1965; Maitland et al., 1997; Woodberry et al., 2008; Kawai et al., 2009; Muh et al., 2018; Muh et al., 2020). A recent study showed highly efficient cross-reactive RhopH2 antibodies against *P. vivax* to *P. knowlesi* which inhibit parasite growth *in vitro* and cross-immunogenicity in clinical samples, thereby highlighting its potential use for cross-protective immunity against both parasites in endemic areas (Muh et al., 2020). The same researchers have shown that in both *P. vivax* and *P. knowlesi*, the apical asparagine (Asn)-rich protein (AARP) antigen has been linked to long-lasting cross-species protective immunity (Muh et al., 2018). Previous studies have also found that *P. falciparum* antigens with structural similarities, such as erythrocyte membrane protein 1 variations and variant surface antigen 2-CSA, *P. vivax* AMA1, and *P. falciparum* AMA1, demonstrated cross-reactivity *via* conserved epitopes (Klein et al., 2008; Drew et al., 2018; Gnidehou et al., 2019).

In this study, we determined the genetic diversity and natural selection acting at the *RhopH2* cross-protective domain (Muh et al., 2020) from *P. knowlesi* as well as *P. vivax* samples: for *P. knowlesi*, 40 samples [37 clinical samples and 3 laboratory lines (along with the H-strain)] from Malaysia, and for *P. vivax*, 65 *PvRhopH2* gene sequences retrieved from clinical samples from 10 countries. We also predicted the cross-species epitopes in *P. vivax* and *P. knowlesi* using bioinformatics tools. Phylogenetic analysis was conducted to understand the relationships between clinical samples and other ortholog species of *Plasmodium* and determine conserved epitope regions. Since this is the first study on *RhopH2* sequences obtained from clinical samples of both species, the results of this study will be helpful in understanding the level of polymorphism within the functional domains in field samples for future functional and strain-transcending vaccine development studies. This will be beneficial for the rational design and formulation of a blood-stage vaccine against *P. knowlesi* and *P. vivax*.

## Materials and Methods

### *PkRhopH2* and *PvRhopH2* Sequence Data

Thirty-seven *PkRhopH2* gene sequences were retrieved from a public database (https://www.ebi.ac.uk/ena/browser/home) from clinical samples originating from Malaysian Borneo and 3 previously isolated lines from Peninsular Malaysia (along with the H-strain PKNH_0727900) (**Supplementary Table 1**) (Assefa et al., 2015). Sixty-five *PvRhopH2* gene sequences were retrieved from clinical samples from 10 countries from PlasmoDB (https://plasmodb.org) (**Supplementary Table 2**) along with 3 reference strains of *P. vivax* (Sal-1; PVX_099930, P01; PVP01_072900 and *P. vivax-*like Pvl01; PVL_000087200). Sequences were aligned using the CLUSTAL-W program in MegAlign Lasergene v 7.0 (DNASTAR), and polymorphism and phylogenetic analyses were conducted in MEGA 5.0 software. In order to determine the relationship between PkRhopH2 sequences (laboratory lines and clinical samples from Sarawak, Malaysian Borneo), phylogenetic analyses were conducted using deduced amino acid sequences using the maximum likelihood (ML) method based on the Poisson correction model as described in MEGA 5.0 with 1,000 bootstrap replicates to test the robustness of the trees. The interspecies phylogenetic analysis was also performed by using the same method in *P. falciparum* (PF3D7_0929400), *P. cynomolgi* (PCYB_073680), *P. coatneyi* (PCOAH_00016180), *P. knowlesi* (PKNH_0727900), and *P. vivax* Sal-1 (PVX_099930). Phylogenetic analysis was also conducted using 65 *PvRhopH2* deduced amino acid sequences and its ortholog species using the same method as used for *P. knowlesi* sequences.

### Epitope Prediction

B cell epitopes are antigenic determinant, portion of foreign protein, or antigen that can be used for developing a peptide vaccine (Saha and Raghava, 2006). In this study, in order to find cross-reactive epitopes between *P. vivax* and *P. knowlesi*, B cell epitopes were predicted *in silico* in RhopH2 amino acid sequences (domain previously studied (Muh et al., 2020) by using the Bcpred server http://www.imtech.res.in/raghava/bcepred/bcepred_team.html (Saha and Raghava, 2006) and the antibody epitope prediction server at the IEDB Analysis resource, by using the Emini Surface Accessibility Prediction model http://tools.immuneepitope.org/bcell (Emini et al., 1985). The Bcpred software predicts B cell epitopes based on amino acid properties, i.e., hydrophilicity, flexibility, polarity, and exposed surface, and a threshold score of 2.38 is considered for epitope prediction. The potential conservation of epitopes between *P. knowlesi*, *P. vivax*, and other primate malaria species was investigated.

### Sequence Diversity and Natural Selection

Sequence diversity (π) was determined by DnaSP v5.10 software (Librado and Rozas, 2009). Number of parsimony informative sites, polymorphic sites, synonymous (silent mutations) and non-synonymous substitution (replacement changes), singletons, number of haplotypes (H), and haplotype and nucleotide diversity within *PkRhopH2* and *PvRhopH2* gene exon 1 (from 64 to 1,161 nt) were also determined by DnaSP software. Nucleotide diversity was also graphically represented using the window length of 100 and step size of 25 bp. The rate of non-synonymous substitution per non-synonymous site (dS) and the rate of synonymous substitution per synonymous site (dN) which determine the natural selection were determined using the method of Nei and Gojobori (1986). Additionally, more analyses were performed to determine natural selection, such as Tajima’s D, Fu and Li’s D*, and F* neutrality tests, which were implemented in DnaSP v5.10 software. Under neutrality, Tajima’s D value should be zero. The negative value of Tajima’s D is indicative of population expansion, and the positive as well as significant value indicates positive selection/balancing. Tajima’s D values were also represented graphically using DnaSP software. Fu and Li’s D^*^ and F* positive and significant values indicate population contraction; singleton excess and negative values indicate population expansion.

## Results

### RhopH2 Sequence Identity and Phylogenetic Relationship Between *P. knowlesi*, *P. vivax*, and Its Ortholog Species

**Figure 1:**
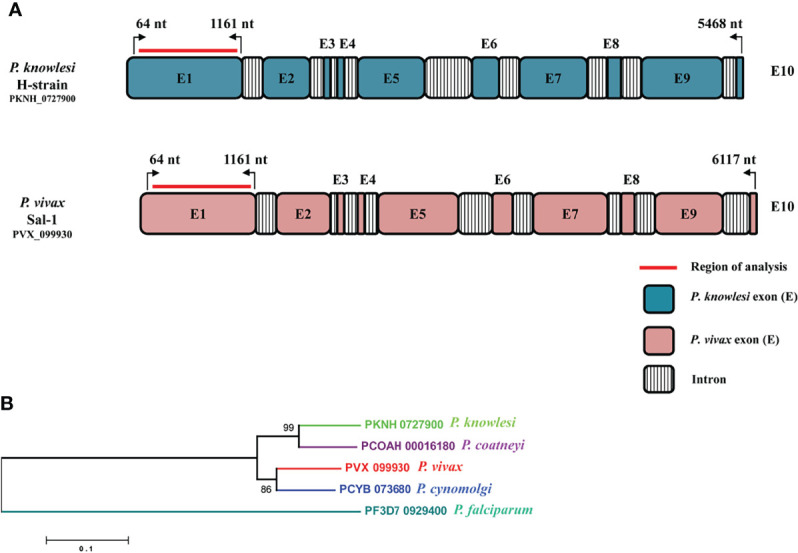
**(A)** Schematic representation of the *P. knowlesi* full-length *RhopH2* gene in H-strain (PKNH_0727900, 5,468 bp) and *P. vivax* sal-1 strain (PVX_099930, 6,117 bp). The introns and exons were determined as described in PlasmoDB (www.plasmodb.org). The number on top with bend arrows represents position of the nucleotides in *P. knowlesi* and in *P. vivax.* The bold red line indicates the region of analysis for this study. **(B)** Interspecies phylogenetic relationships between RhopH2 full-length amino acid sequences of orthologs i.e., *P. knowlesi* (PKNH_0727900), *P. coatneyi* (PCOAH_00016180), *P. vivax* (PVX_099930), *P*. *cynomolgi* (PCYB_073680), and *P. falciparum* (PF3D7_0929400).

The amino acid sequence identity of the RhopH2 region (64 to 1,161 nt, Exon I) which exhibited high cross-reactivity (Muh et al., 2020) with the *P. knowlesi* H-strain and *P. vivax* Sal I was found to be 74.44%. A schematic diagram of the full-length 10-exon structure of the *RhopH2* gene of *P. knowlesi* in comparison to *P. vivax* sal-1 is shown in **Figure 1A**. A conserved 10-exon structure was observed within both species with length variations in Exons II, V, VII, and IX in *P. vivax* (**Figure 1A**). The phylogenetic analysis performed using deduced amino acid sequences in the ML method showed that PkRhopH2 is more closely related with *P. coatneyi* in comparison to its other orthologs in *P. vivax*, *P. cynomolgi*, and *P. falciparum* (**Figure 1B**). However, no geographical clustering was noted for *P. vivax* samples originating from 10 countries.

### Genetic Diversity and Polymorphisms of *PkRhopH2* in Clinical Samples

**Figure 2:**
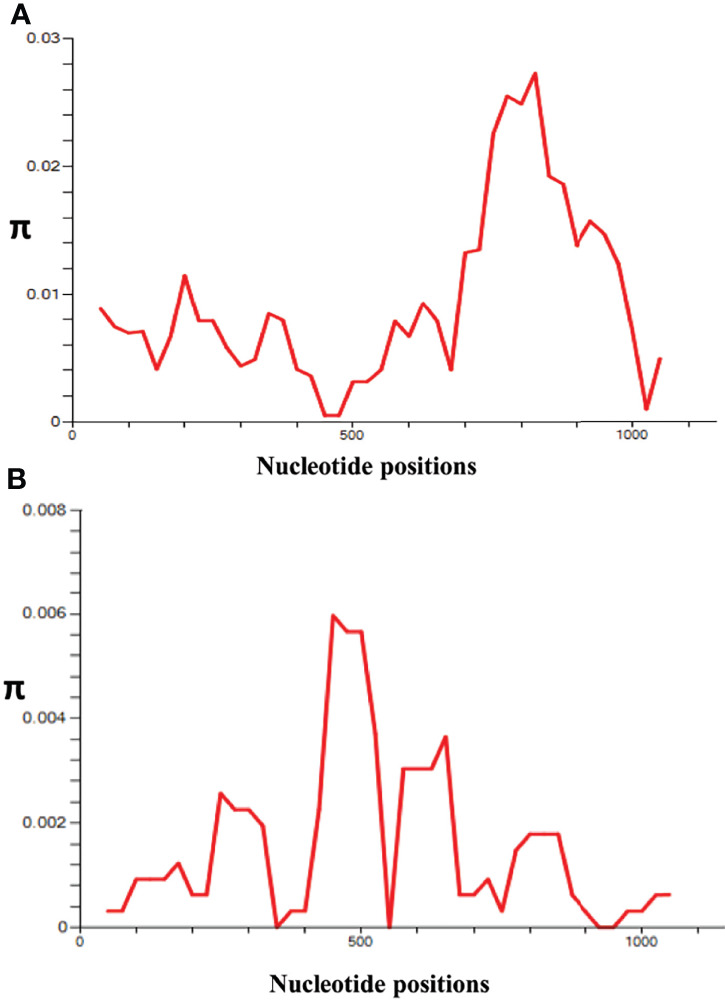
l **(A)** Graphical representation of nucleotide diversity (π) within the *PkRhopH2* gene showing high diversity in the region from 700 to 900 nt. **(B)** Graphical representation of nucleotide diversity (π) within the *PvRhopH2* gene showing high diversity in the region from 400 to 550 nt. The window length and step size of the π graph are 100 and 25, respectively, as implemented in DnaSP software v5.0.

The nucleotide alignment of 40 *PkRhopH2* sequences revealed that there were 49 single-nucleotide polymorphisms (SNPs) (**Figure S1**), of which 24 were synonymous substitutions and 22 non-synonymous substitutions. The overall nucleotide diversity was found to be π = 0.00936 which was higher compared to *PvRhopH2*; π = 0.00147 (**Table 1**). Analysis of 65 *PvRhopH2* sequences revealed 23 SNPs (14 were synonymous substitutions and 9 non-synonymous substitutions). Twenty-three *PvRhopH2* SNPs observed within 65 sequences are shown in **Figure S5**. *PkRhopH2* had 38 parsimony informative sites out of which three were tri-variants, 8 singleton variable sites, 34 haplotypes with the haplotype diversity of Hd = 0.988 (**Table 1**). *PvRhopH2* sequences revealed 23 singleton sites, 6 parsimony informative sites, and 9 haplotypes with haplotype diversity of Hd = 0.548 (**Table 1**). The graphical representation of the nucleotide diversity for both species is shown in **Figures 2A, B**, respectively. Graphical representations of Tajima’ D values are shown in **Figures S4A, B**. The amino acid sequence alignment of 40 PkRhopH2 sequences identified 3 sites with triple variants (T225S/N, Q281R/K, V302S/A) (**Figure S2**). The amino acid sequence alignment of 65 PvRhopH2 sequences with 9 non-synonymous substitutions is shown in **Figure S6**.

**Table 1:** Estimates of nucleotide diversity, haplotype diversity, and neutrality indices of *P knowlesi* and *P. vivax* RhopH2 genes.

Domain	No. of samples	SNPs	Syn	Non-syn	No. of haplotypes	Diversity ± SD		Taj D	Fu and Li’s D*	Fu and Li’s F*
						Haplotype	Nucleotide			
*PkRhopH2*	40	49	24	22	34	0.988 ± 0.010	0.00936 ± 0.0013	-0.382 *p* > 0.10	0.61568 *p* > 0.10	0.31781 *p* > 0.10
*PvRhopH2*	65	23	14	09	9	0.548 ± 0.070	0.00147 ± 0.0004	-2.081 *p* < 0.05	-4.342 *p* < 0.02	-4.197 *p* < 0.02

SNPs, single-nucleotide polymorphisms; SD, standard deviation; Syn, synonymous substitutions; non-syn, non-synonymous substitutions; NA, not applicable.

P. vivax results by using MEGA 5.0 software.

**Figure 3:**
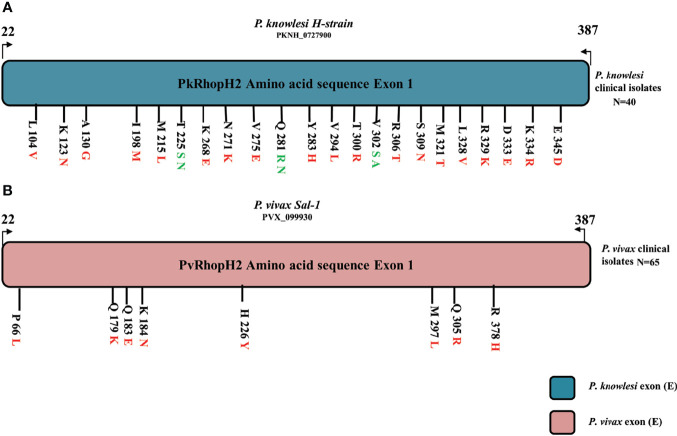
**(A)** Schematic representation of amino acid polymorphism in clinical isolates in *P. knowlesi* RhopH2 protein. Amino acid polymorphism is represented by black lines with letter and numbers. The letter before number represents the reference H-strain amino and polymorphism is represented in red (two variants) and green color (three variants). Similarly in *P. vivax* clinical samples in **(B).** The alphabet letter represents the amino acid one-letter code, and numbers represent amino acid position.

The schematic representation of 22 non-synonymous substitutions observed within 40 samples with reference to *P. knowlesi* reference H strain is shown in **Figure 3A**. Similarly, the *P. vivax* non-synonymous substitutions within 65 samples with reference to the Sal-1 strain are shown in **Figure 3B**.

### Natural Selection in *PkRhopH2* and *PvRhopH2*

The natural selection analysis of the *RhopH2* gene from 40 sequences indicated that the gene is under negative or purifying selection (dS-dN = 2.41, p < 0.009) probably due to functional constraints (**Table 1**). We found a similar strong negative selection acting at the *PvRhopH2* domain (dS-dN = 1.59, p < 0.05). The overall Tajima’s D value was negative for both species (*PkRhopH2*; D = -0.38, p > 0.05 and *PvRhopH2*; D = -2.08, p < 0.05), which indicates purifying selection and population expansion. Fu and Li’s D* and F^*^ values were positive (0.618 and 0.317) but not significant for *PkRhopH2*. Significant values were obtained for *PvhopH2* -4.32 and -4.17, respectively (**Table 1**).

### Phylogenetic Analysis

**Figure 4:**
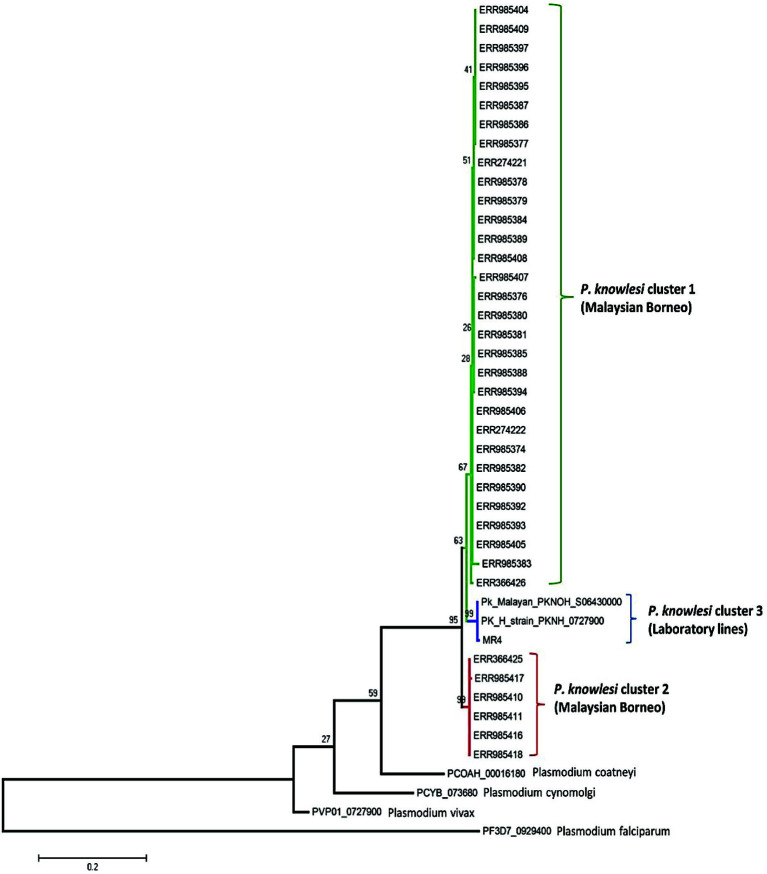
Phylogenetic tree of PkRhopH2 proteins (cross-protective domain, amino acid positions 22–387) from clinical samples of Malaysia and its orthologs in other *Plasmodium* species is constructed based on the maximum likelihood method. Cluster 1 and cluster 2 represent the two subpopulations of clinical samples from Malaysian Borneo, and the 3^rd^ cluster contains lab strains of *P. knowlesi*. Bootstrap values are indicated by numbers at nodes.

The phylogenetic analysis of the 40 samples of PkRhopH2 amino acid sequences with its ortholog species in *Plasmodium* by using the maximum likelihood method identified three different population clusters or subpopulation (Cluster 1, Cluster 2, and Cluster 3) (**Figure 4**). Out of these three clusters, two clusters originated from Malaysian Borneo and cluster 3 belonged to laboratory lines containing the H-strain. These clusters were linked to the primary hosts of *P. knowlesi* which are *Macaca nemestrina* (cluster 1) and *Macaca fascicularis* (cluster 2) as previously reported (Divis et al., 2015; Pinheiro et al., 2015; Ahmed et al., 2016; Ahmed et al., 2018a). The phylogenetic analysis of 65 PvRhopH2 amino acid sequences with its ortholog species in *Plasmodium* using the ML method revealed that there was no geographical clustering (**Figure S7**).

### B Cell Epitopes in PkRhopH2

**Figure 5:**
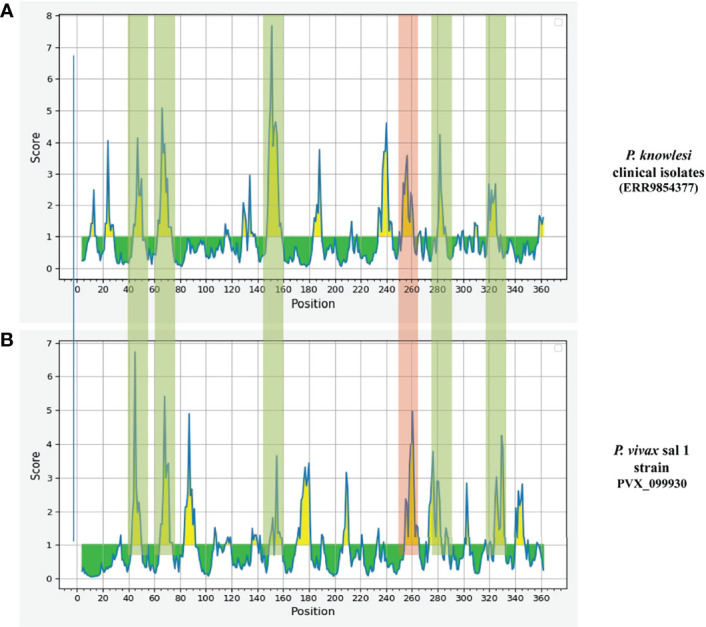
The graphs (**A** *P. knowlesi* and **B** *P. vivax*) are obtained from epitope prediction software IEDB. The graph represents the possible epitope region in RhopH2 protein. The peaks in graph show the possible epitope region in RhopH2 protein. The highlighted part represents the overlapping of the peaks in *P. knowlesi* and *P. vivax.* This indicates there is possibility of epitopes which have one or more common amino acids. The peak highlighted in red regions shows a 100% conserved amino acid sequence in all three clusters of *P. knowlesi* clinical isolate and *P. vivax*.

Epitope prediction using Bcpred and IEDB servers identified 11 epitopes in *P. knowlesi* RhopH2 (**Table 2A** and **Figure 5A**). In *P. vivax*, Bcpred and IEDB servers identified 11 and 9 RhopH2 epitopes, respectively (**Table 2B** and **Figure 5B**). The comparison of epitope outputs from both servers revealed a total of 10 P*. knowlesi* RhopH2 epitopes ranging in length from 6 to 15 amino acids (**Table 2A**), while 9 P*. vivax* RhopH2 epitopes were identified by both servers (**Table 2B**). The interspecies comparison of epitopes identified a highly conserved epitope comprising of 10 amino acid (256QNSKHFKKEK265) (**Tables 2A, B**). This conservation of epitope was also observed in all 3 P*. knowlesi* clusters which were identified by phylogenetic analysis as well as 62 clinical samples of *P. vivax* from 10 countries. However, in comparison with the conserved epitope region found in *P. knowlesi* and *P. vivax*, there was a difference of 2 amino acids (256ENSKHFKKDK265) in its simian orthologs, i.e., *P. coatneyi* and *P. cynomolgi* and 1 amino acid difference (256QNSKHFKKDK265), respectively (**Figure S3**). Analysis of diversity and prevalence of the 10 PkRhopH2 epitopes (common epitopes identified by both software) in clinical samples indicated that 7 epitopes (70%) were 100% conserved (**Table 3**). The remaining 3 epitopes had at least 2–4 variants (**Table 3**). Analysis of diversity and prevalence of 9 PvRHopH2 epitopes (common epitopes identified by both software) from 65 samples from 10 countries indicated that 7 epitopes (77%) were 100% conserved **(** **Table 4**).

**TABLE 2(A):** The possible epitope predicted by using the IEDB server and Bcpred server in *P. knowlesi* shown in the above table.

IEDB server				Bcpred server				
No.	Start AA	End AA	Peptide	Length	Start AA	End AA	Peptide	Length
1	31	36	KNTPDA	6	-	-	-	-
2	43	49	**VENDKNK**	7	42	53	Q**VENDKNK**ICKN	12
3	65	71	S**QNEEDS**	7	66	72	**QNEEDS**Y	7
4	84	94	**KNDTPNE**TTEA	11	84	90	**KNDTPNE**	7
5	154	159	**RSSVKN**	6	148	158	NRFIKD**RSSVK**	11
6	169	179	**KEDEYTNKAKQ**	11	167	181	SS**KEDEYTNKAKQ**NM	15
7	204	210	**KVPKRYS**	7	202	213	TV**KVPKRYS**AEN	12
8	255	265	**DQNSKHFKKEK**	11	256	268	**QNSKHFKKEK**LLE	13
9	273	282	**EYELDKESRI**	10	273	283	**EYELDKESRI**Y	11
10	301	307	**DSNGKRK**	7	301	311	**DSNGKRK**LSVR	11
11	340	347	**KNLRRELN**	8	338	349	TM**KNLRRELN**DE	12
12	–	–	–	NA	381	387	DYEDIEK	7

The common amino acids within an epitope predicted by both servers are indicated in bold. Amino acids in red indicate the conserved epitope among all species.

**Table 2(B):** The epitope predicted by using the IEDB server and Bcpred server in *P. vivax* shown in the above table.

IEDB server					Bcpred server			
No.	Start AA	End AA	Peptide	Length	Start AA	End AA	Peptide	Length
1	43	50	**VEKDKKKI**	8	41	54	IE**VEKDKKKI**CKNA	14
2	65	74	**SPREEETYVQ**	10	65	75	**SPREEETYVQ**K	11
3	83	91	I**KNDSPDE**S	9	84	90	**KNDSPDE**	7
4	–	–	–	–	137	145	ALKRAKQLI	9
5	154	159	**KAKVKN**	6	148	162	NRFIKD**KAKVKN**VQE	15
6	171	181	DDF**MNEPKQKM**	11	174	184	**MNEPKQKM**LQK	11
7	–	–	–	–	207	213	KRYSSET	7
8	255	265	**DQNSKHFKKEK**	11	256	268	**QNSKHFKKEK**LLE	13
9	274	282	**YRVNRESKV**	9	273	284	D**YRVNRESKV**HE	12
10	324	332	**RDIEKREIS**	9	323	334	G**RDIEKREIS**ER	12
11	341	347	**NLRKDLN**	7	338	349	TVK**NLRKDLN**DE	12

The common epitopes predicted by both servers are indicated in bold, and the conserved epitopes among all species as mentioned in results are indicated in red color.

AA, amino acid.

**Table 3:** Diversity of the common epitopes predicted by both servers and their prevalence in clinical isolates of *P. knowlesi*.

No.	Start AA	End AA	Peptide	Length	(%)
1	43	49	**VENDKNK**	7	100
2	65	71	S**QNEEDS**	7	100
3	84	94	**KNDTPNE**	11	100
4	154	159	**RSSVKN**	6	100
5	169	179	**KEDEYTNKAKQ**	11	100
6	204	210	**KVPKRYS**	7	100
7	255	265	**QNSKHFKKEK**	11	100
8	273	282	**EYELDKESRI**	10	77.5
			**EYELDKESKI**		15
			**EYVLDKESQI**		7.5
9	301	307	**DSNGKRK**	7	75
			**DANGKRK**		2.5
			**DVNGKTK**		15
			**DVNGKRK**		7.5
10	340	347	**KNLRRELN**	8	45
			**KNLRRDLN**		55

Amino acids in red indicate polymorphism within epitopes.

**Table 4:** Diversity of the common epitopes predicted by both servers and their prevalence in 65 clinical isolates of *P. vivax RhopH2*.

No.	Start AA	End AA	Peptide	Length	(%)
1	43	50	**VEKDKKKI**	8	100
2	65	74	**SPREEETYVQ**	10	98.5
			**SLREEETYVQ**		1.5
3	84	90	**KNDSPDE**	7	100
4	154	159	**KAKVKN**	6	100
5	174	181	**MNEPKQKM**	8	89.2
			**MNEPKKKM**		10.08
6	256	265	**QNSKHFKKEK**	10	100
7	274	282	**YRVNRESKV**	9	100
8	324	332	**RDIEKREIS**	9	100
9	340	347	**NLRKDLN**	7	100

Amino acids in red indicate polymorphism within epitopes.

## Discussion

Antigens which are expressed during blood stages of the malaria parasite’s life cycle, specifically during the merozoite invasion process, e.g., micronemes and rhoptries, are excellent candidates for vaccine development as they are exposed to host immune response. An ideal vaccine candidate is expected to possess low levels of polymorphism but high and long-lasting antigenicity, along with strain-transcending efficacy across different geographical locations. We studied the genetic diversity and natural selection and predicted the B cell epitopes of a RhopH2 domain in *P. knowlesi* and *P. vivax* which has previously shown cross-species immunity (Muh et al., 2020).

Recently, the role of cross-species protective immunity has been reported using apical asparagine (Asn)-rich protein (AARP) in *P. vivax* and *P. knowlesi* (Muh et al., 2018). In this study, the overall nucleotide diversity of *PvRhopH2* and *PkRhopH2* was found to be low (π ~ 0.0014 and 0.009, respectively). These diversity values were lower than a previously reported cross-species candidate AARP, indicating that RhopH2 can be an excellent vaccine candidate as the antigen also showed growth inhibitory as well as cross-species reactive immunity (Muh et al., 2020). The amino acid sequence identity of RhopH2 between the *P. knowlesi* H-strain and *P. vivax* Sal I was found to be 74.44% which was similar to findings of AARP (Muh et al., 2018). Tests of natural selection for both species indicated strong purifying selection probably due to functional constrains in the cross-protective domain studied here; however, Taj’s D and Li and Fu’s D* and F* values were positive for *P. knowlesi* but not significant. This is probably due to existence of *P. knowlesi* subpopulations (Emini et al., 1985; Assefa et al., 2015; Ahmed et al., 2016; Ahmed et al., 2018b). The graphical representation of the nucleotide diversity was high from nucleotide positions 765 to 810. The overall average Tajima’s D value was found out to be negative, but it was positive in the regions of high diversity indicating probable epitope regions.

The ML phylogenetic tree identified 3 subpopulations of PkRhopH2, cluster 1 and cluster 2 from Malaysian Borneo and cluster 3 comprising only the laboratory lines as observed in other invasion genes and population genomic studies (Assefa et al., 2015; Pinheiro et al., 2015; Ahmed et al., 2016; Yusof et al., 2016; Divis et al., 2017; Ahmed et al., 2018a; Ahmed et al., 2018b; Ahmed et al., 2018c; Ahmed et al., 2019).

In this study, we also investigated whether shared epitopes are present in both species in the RhopH2 domain where high cross reactivity and immunogenicity have been observed by Muh et al. (2020). Among multiple epitopes identified by both the software (Bcpred server and IEDB server), a conserved epitope comprising 10 amino acids (QNSKHFKKEK) was found in both *P. knowlesi* and *P. vivax* clinical samples, indicating the possible reason for high cross-species reactivity and immunogenicity as observed by Muh et al. (2020). Interestingly, Taj’s D values around the epitope region gave a positive peak which may further confirm the prediction. This conserved epitope region was also present in all the three clusters of *P. knowlesi* obtained through phylogenetic analysis, which could indicate high antigenicity in clinical samples. This Rhoph2 epitope was also found in other simian malaria parasites, i.e., *P. cynomolgi* and *P. coatneyi*, which showed 80%–90% conservation indicating the possibility of cross-species reactivity and immunogenicity; however, further studies need to be conducted to understand the functional aspect of these epitopes. *P. falciparum* antigens with structural similarities, such as erythrocyte membrane protein 1 variations and variant surface antigen 2-CSA, *P. vivax* AMA1, and *P. falciparum* AMA1, demonstrated cross-reactivity *via* conserved epitopes (Klein et al., 2008; Gnidehou et al., 2019). The results obtained in this study thus provide further supportive evidence for the existence of cross-protective immunity between *P. vivax* and *P. knowlesi* conferred through a shared common epitope. This could serve as a vaccination strategy to protect Southeast Asian residents from *P. knowlesi* infections. To our knowledge, this is the first study to identify novel RhopH2 epitopes and genetic characterization in both species, i.e. *P. knowlesi* and *P. vivax*, thereby contributing significantly toward new knowledge and understanding of the cross-species epitopes for vaccine development. Through our study, the functional role of antibodies specific to the novel conserved epitope identified in this study can be explored for protection against malaria infection.

## Data Availability Statement

Publicly available datasets were analyzed in this study. The repository is ENA (European nucleotide archive https://www.ebi.ac.uk/ena/browser/home), and accession numbers can be found in the **Supplementary Material**.

## Author Contributions

MAAh participated in the conception, design of the study, data collection, analysis, interpretation, and manuscript preparation. GD, RZ, AS, MAAl, SP, and SW participated in the laboratory procedures, data collection, and analysis and manuscript preparation. All authors contributed to the article and approved the submitted version.

## Funding

This study was funded by the Department of Biotechnology, Govt. of India, No.BT/RLF/Re-entry/09/2017, Deanship of Scientific Research at Najran University, Najran, Saudi Arabia NU/IFC/ENT/01/007, Ministry of Health & Welfare, Republic of Korea (HV20C0142) and the National Research Foundation of Korea (NRF) (2018R1A6A1A03025124).

## Conflict of Interest

The authors declare that the research was conducted in the absence of any commercial or financial relationships that could be construed as a potential conflict of interest.

## Publisher’s Note

All claims expressed in this article are solely those of the authors and do not necessarily represent those of their affiliated organizations, or those of the publisher, the editors and the reviewers. Any product that may be evaluated in this article, or claim that may be made by its manufacturer, is not guaranteed or endorsed by the publisher.
