# Supplementary figures and images for: Identification, Mapping, and Genetic Diversity of Novel Conserved Cross-Species Epitopes of RhopH2 in Plasmodium knowlesi With Plasmodium vivax
Source: Front Cell Infect Microbiol. 2022 Jan 13;11:810398. doi: 10.3389/fcimb.2021.810398 (PMC8793677; doi:10.3389/fcimb.2021.810398)

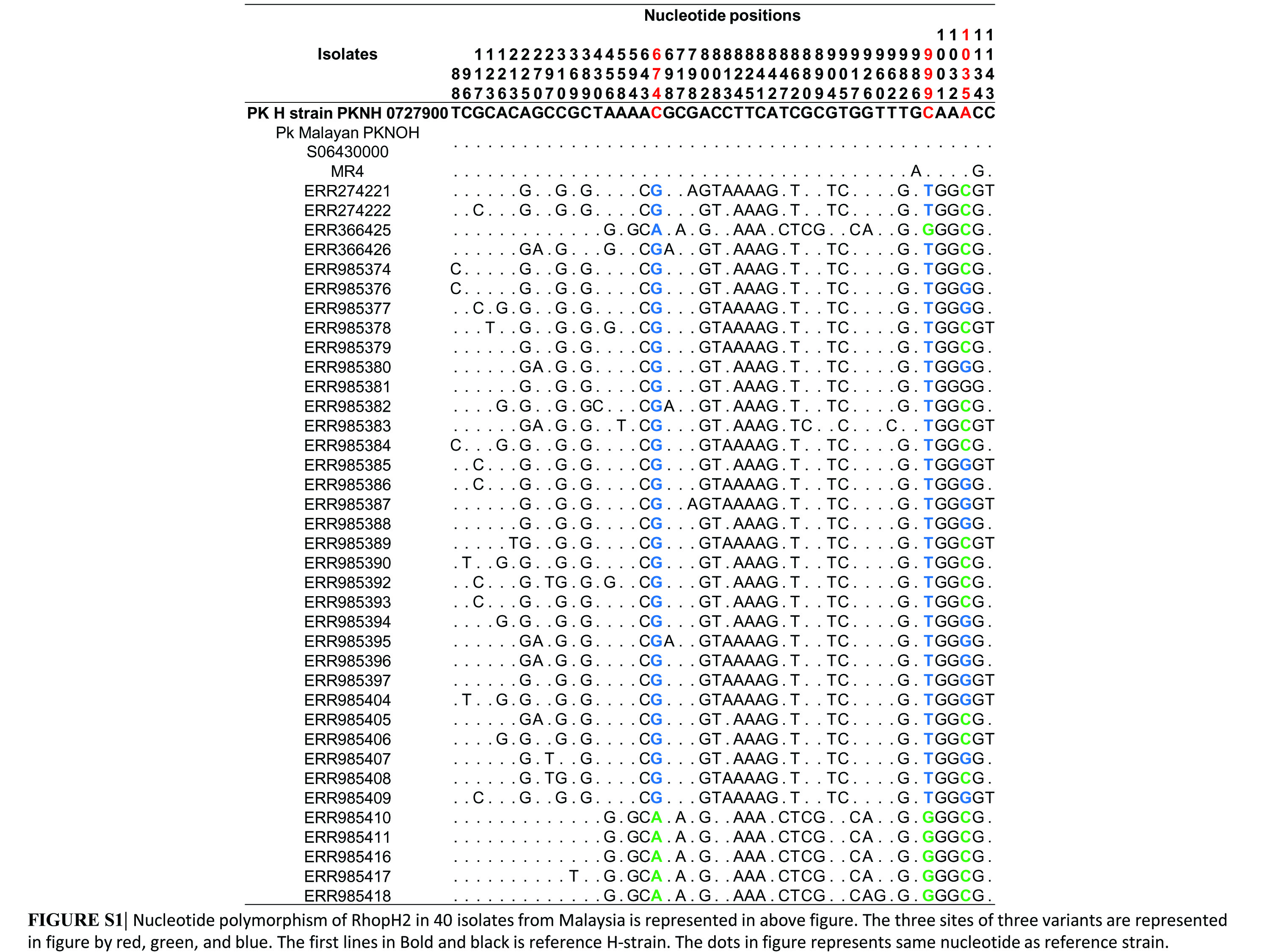

Supplement: Supplementary file 1 [file Image_1.jpeg]

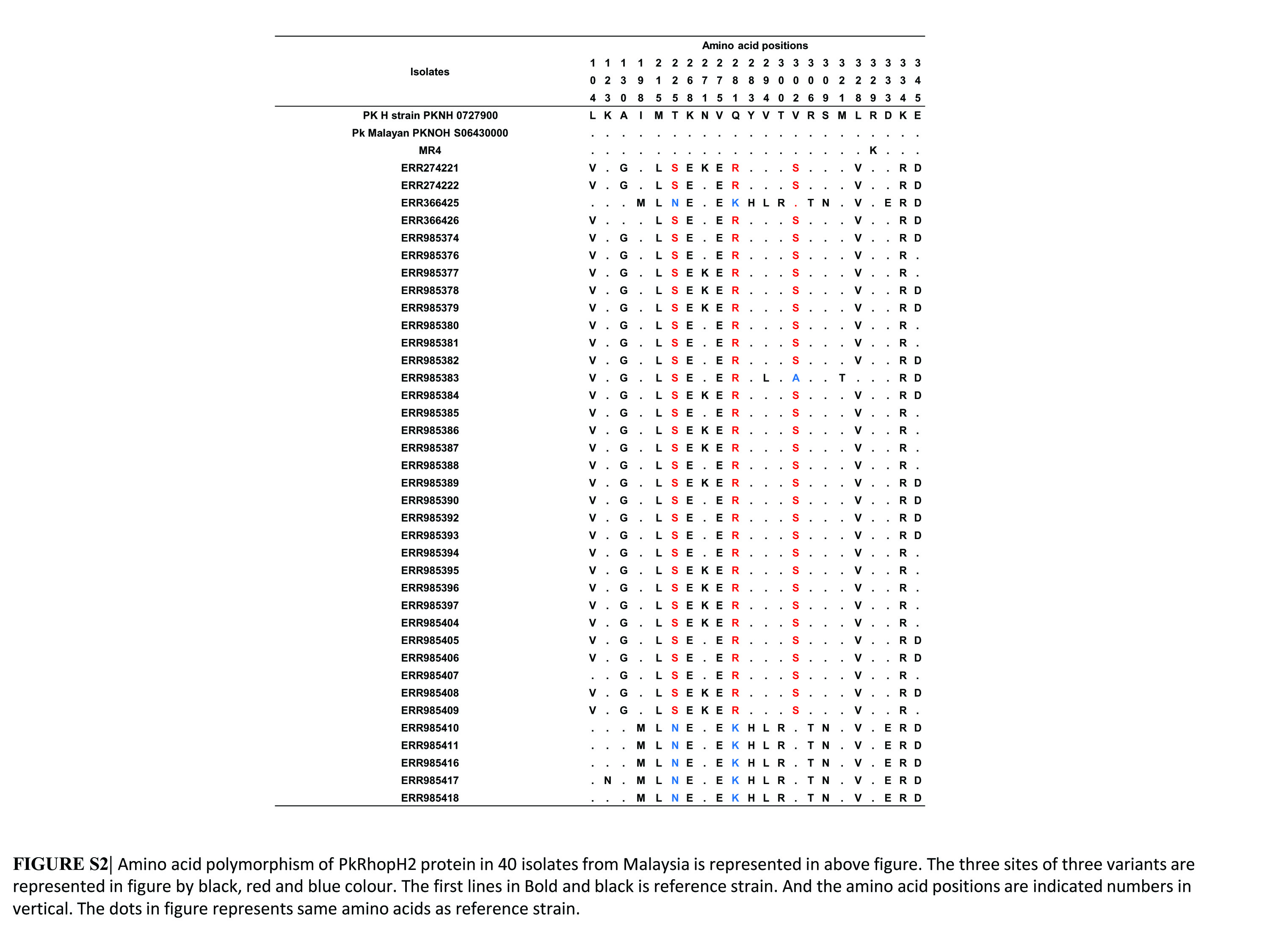

Supplement: Supplementary file 2 [file Image_2.jpeg]

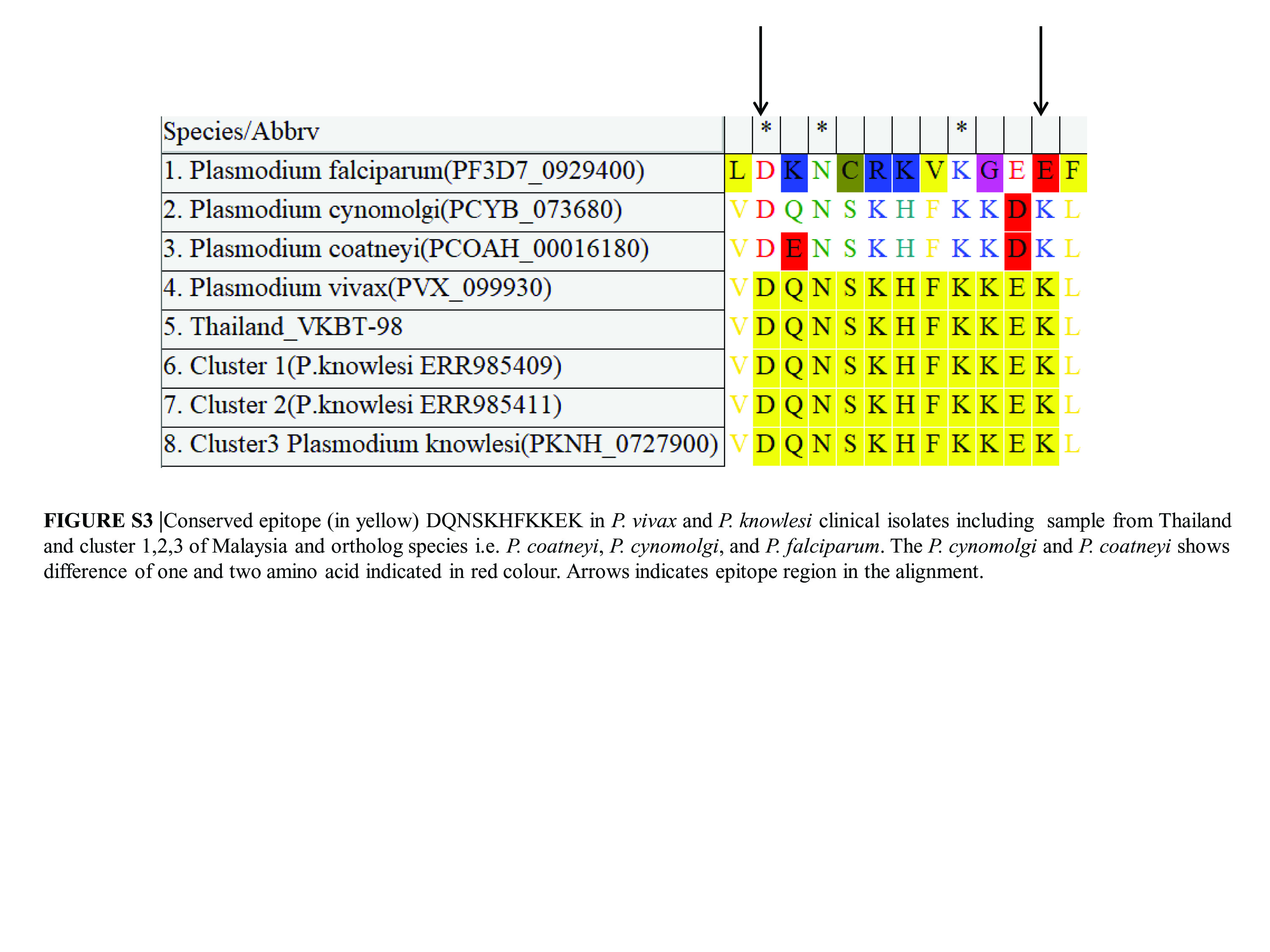

Supplement: Supplementary file 3 [file Image_3.jpeg]

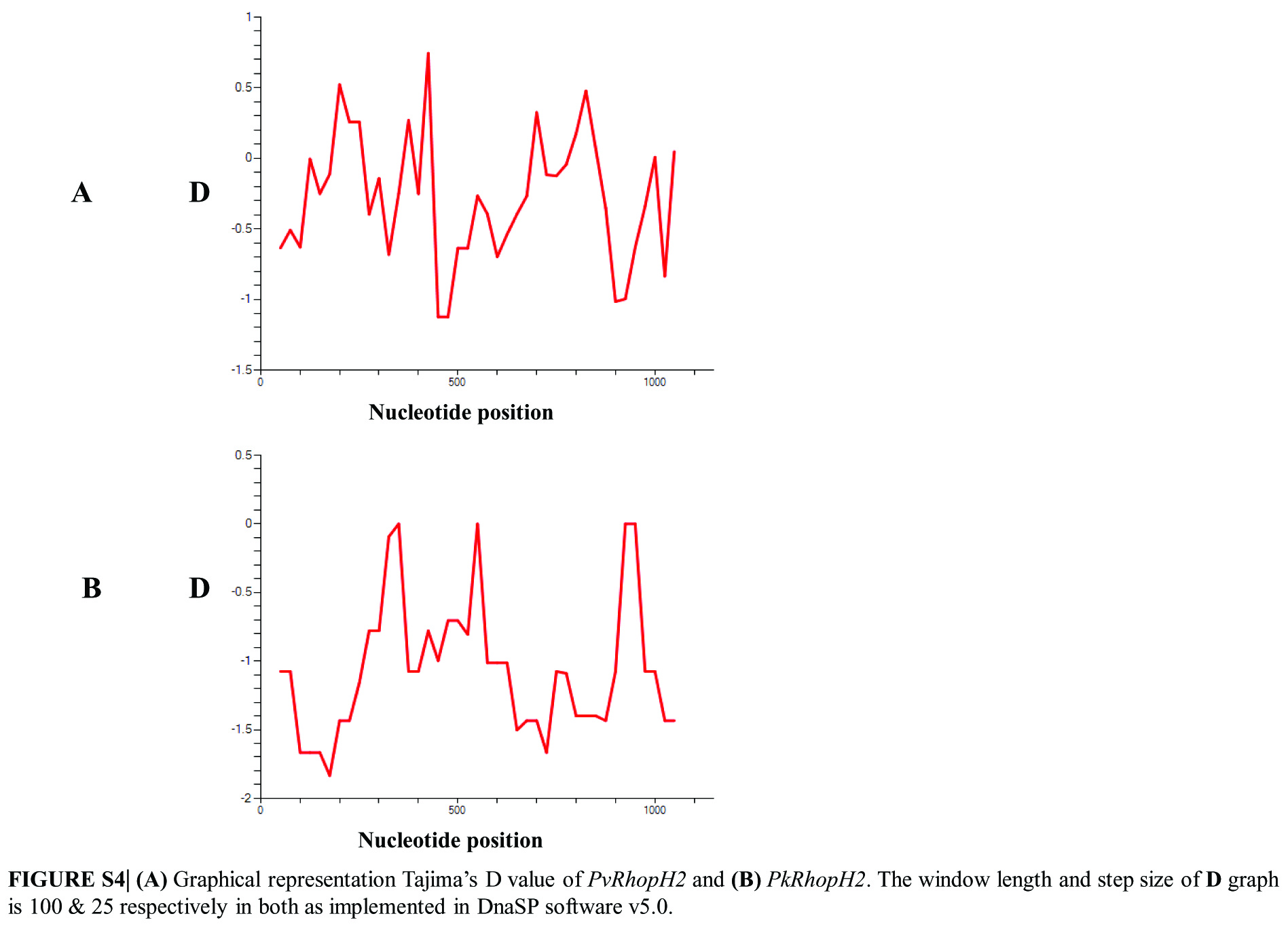

Supplement: Supplementary file 4 [file Image_4.jpg]

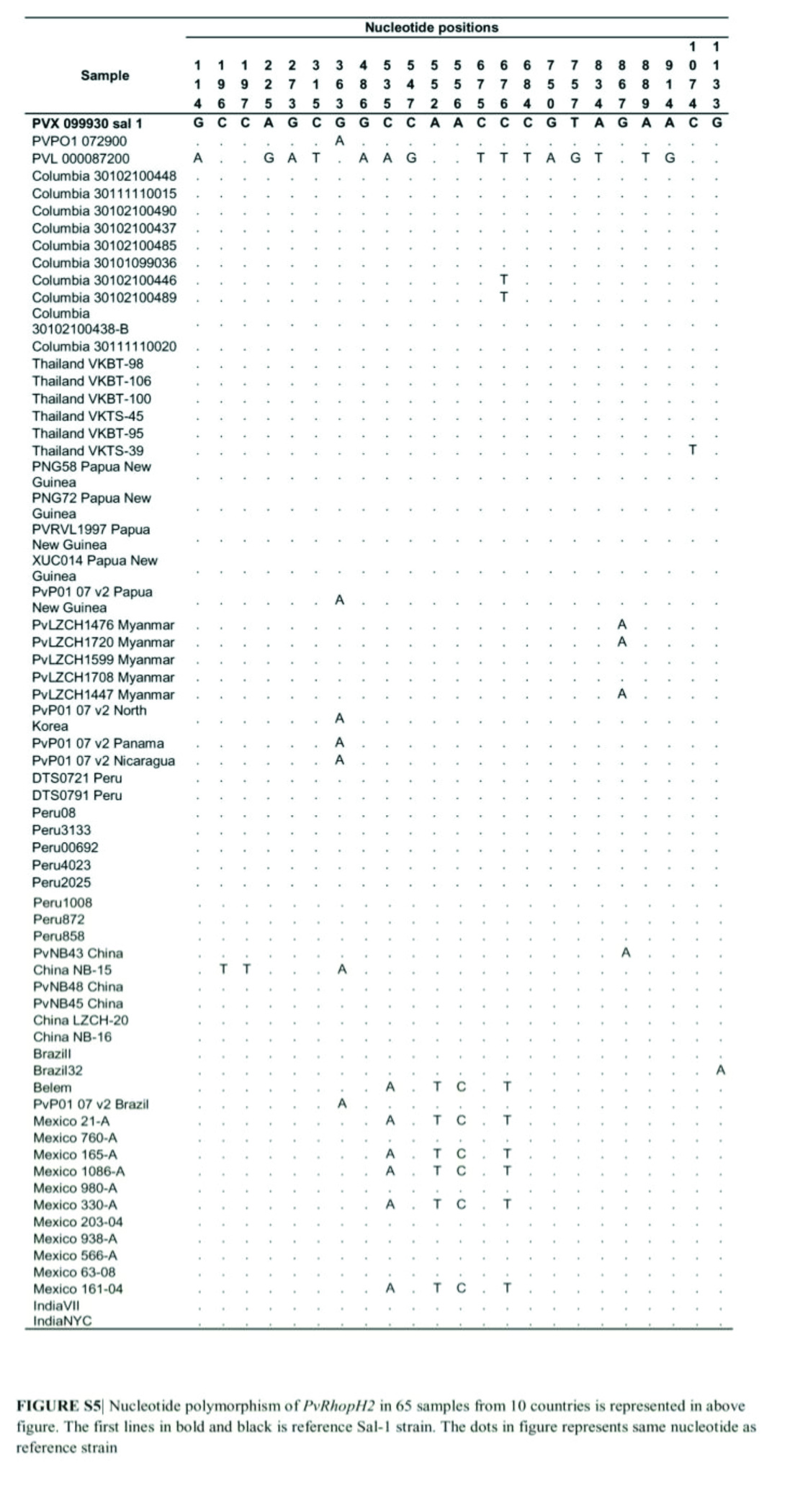

Supplement: Supplementary file 5 [file Image_5.jpeg]

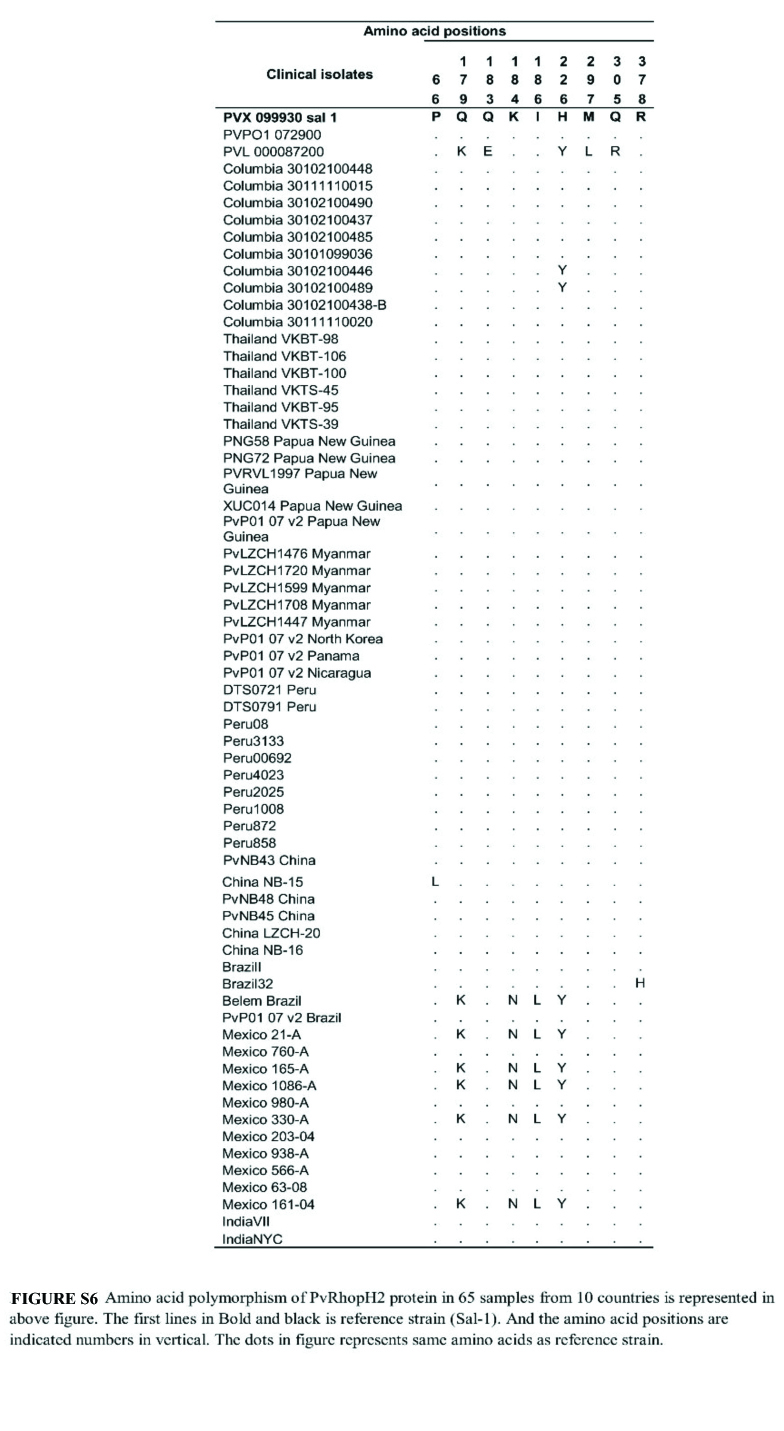

Supplement: Supplementary file 6 [file Image_6.jpeg]

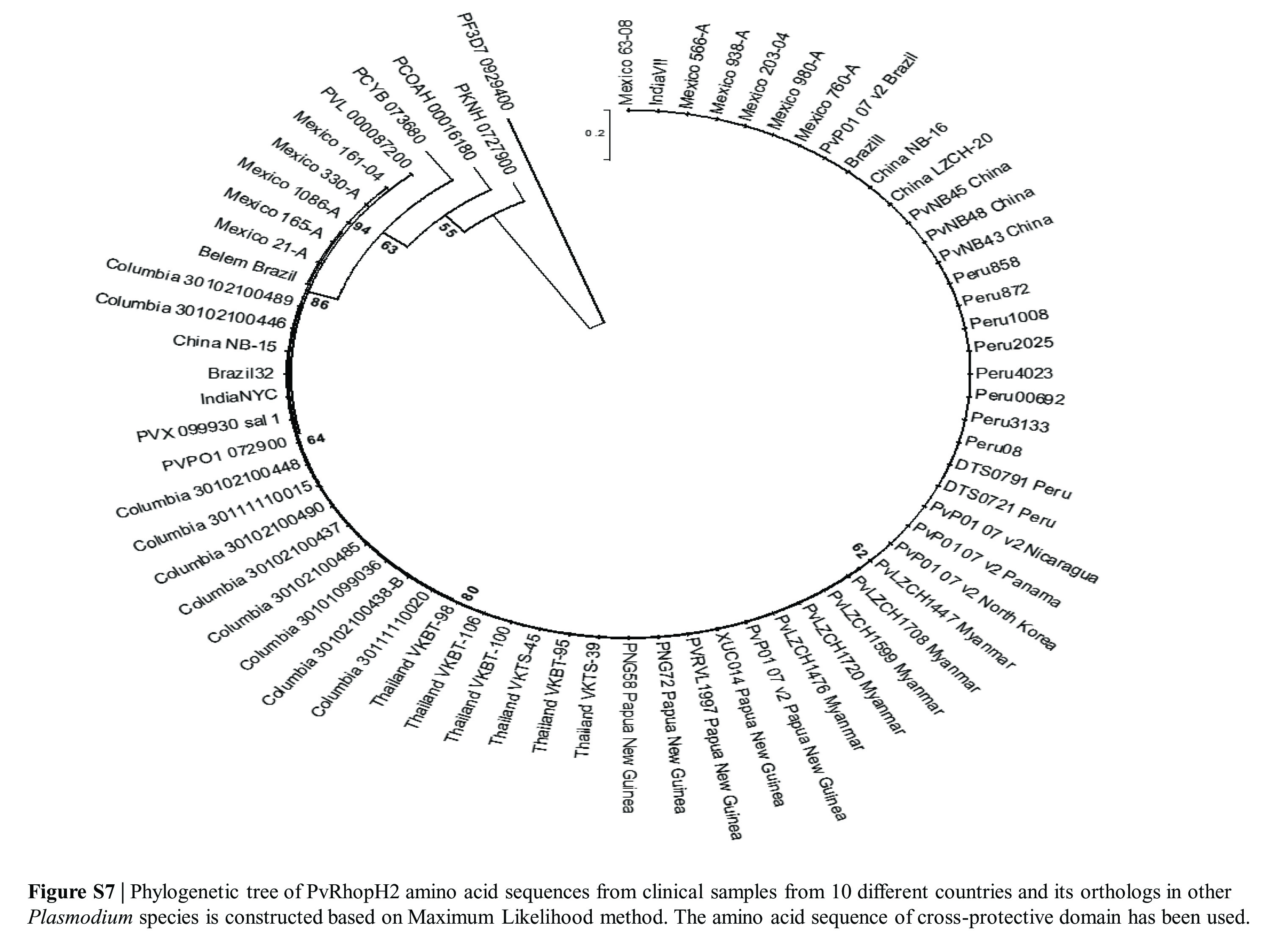

Supplement: Supplementary file 7 [file Image_7.jpeg]
