# Supplementary material for: Identification, Mapping, and Genetic Diversity of Novel Conserved Cross-Species Epitopes of RhopH2 in Plasmodium knowlesi With Plasmodium vivax
Source: Front Cell Infect Microbiol. 2022 Jan 13;11:810398. doi: 10.3389/fcimb.2021.810398 (PMC8793677; doi:10.3389/fcimb.2021.810398)
Supplement: Supplementary file 8 [file Table_1.docx]

| **Supplementary Table 1. *PkRhopH2* Study samples and origin** | | |
| --- | --- | --- |
| **No.** | **Accession number** | **Geographical Location** |
|  |  |  |
| 1 | PKNH_0727900 | Peninsular malaysia |
| 2 | PKNOH_S06430000 | Peninsular malaysia |
| 3 | MR4 | Peninsular malaysia |
| 4 | ERR274221 | Sarikei, Malaysian Borneo |
| 5 | ERR274222 | Sarikei, Malaysian Borneo |
| 6 | ERR366425 | Sarikei, Malaysian Borneo |
| 7 | ERR364226 | Sarikei, Malaysian Borneo |
| 8 | ERR985374 | Betong, Malaysian Borneo |
| 9 | ERR985376 | Betong, Malaysian Borneo |
| 10 | ERR985377 | Betong, Malaysian Borneo |
| 11 | ERR985378 | Betong, Malaysian Borneo |
| 12 | ERR985379 | Betong, Malaysian Borneo |
| 13 | ERR985380 | Betong, Malaysian Borneo |
| 14 | ERR985381 | Betong, Malaysian Borneo |
| 15 | ERR985382 | Betong, Malaysian Borneo |
| 16 | ERR985383 | Betong, Malaysian Borneo |
| 17 | ERR985384 | Betong, Malaysian Borneo |
| 18 | ERR985410 | Betong, Malaysian Borneo |
| 19 | ERR985411 | Betong, Malaysian Borneo |
| 20 | ERR985385 | Kapit, Malaysian Borneo |
| 21 | ERR985386 | Kapit, Malaysian Borneo |
| 22 | ERR985387 | Kapit, Malaysian Borneo |
| 23 | ERR985388 | Kapit, Malaysian Borneo |
| 24 | ERR985389 | Kapit, Malaysian Borneo |
| 25 | ERR985390 | Kapit, Malaysian Borneo |
| 26 | ERR985392 | Kapit, Malaysian Borneo |
| 27 | ERR985393 | Kapit, Malaysian Borneo |
| 28 | ERR985394 | Kapit, Malaysian Borneo |
| 29 | ERR985395 | Kapit, Malaysian Borneo |
| 30 | ERR985396 | Kapit, Malaysian Borneo |
| 31 | ERR985397 | Kapit, Malaysian Borneo |
| 32 | ERR985404 | Kapit, Malaysian Borneo |
| 33 | ERR985405 | Kapit, Malaysian Borneo |
| 34 | ERR985406 | Kapit, Malaysian Borneo |
| 35 | ERR985407 | Kapit, Malaysian Borneo |
| 36 | ERR985408 | Kapit, Malaysian Borneo |
| 37 | ERR985409 | Kapit, Malaysian Borneo |
| 38 | ERR985416 | Kapit, Malaysian Borneo |
| 39 | ERR985417 | Kapit, Malaysian Borneo |
| 40 | ERR985418 | Kapit, Malaysian Borneo |
|  |  |  |
