# Supplementary material for: Identification, Mapping, and Genetic Diversity of Novel Conserved Cross-Species Epitopes of RhopH2 in Plasmodium knowlesi With Plasmodium vivax
Source: Front Cell Infect Microbiol. 2022 Jan 13;11:810398. doi: 10.3389/fcimb.2021.810398 (PMC8793677; doi:10.3389/fcimb.2021.810398)
Supplement: Supplementary file 9 [file Table_2.docx]

|  | |  |  |
| --- | --- | --- | --- |
| **Supplementary Table 2. *P. vivax RhopH2 s*tudy samples and origin** | | | |
| **No.** | **Sample ID's** | | **Geographical Location** |
| 1 | **PVX 099930 sal 1** | | - |
| 2 | PVPO1 072900 | | - |
| 3 | PVL 000087200 | | - |
| 4 | Columbia 30102100448 | | columbia |
| 5 | Columbia 30111110015 | | columbia |
| 6 | Columbia 30102100490 | | columbia |
| 7 | Columbia 30102100437 | | columbia |
| 8 | Columbia 30102100485 | | columbia |
| 9 | Columbia 30101099036 | | columbia |
| 10 | Columbia 30102100446 | | columbia |
| 11 | Columbia 30102100489 | | columbia |
| 12 | Columbia 30102100438-B | | columbia |
| 13 | Columbia 30111110020 | | columbia |
| 14 | Thailand VKBT-98 | | Thailand |
| 15 | Thailand VKBT-106 | | Thailand |
| 16 | Thailand VKBT-100 | | Thailand |
| 17 | Thailand VKTS-45 | | Thailand |
| 18 | Thailand VKBT-95 | | Thailand |
| 19 | Thailand VKTS-39 | | Thailand |
| 20 | PNG58 Papua New Guinea | | Papua New Guinea |
| 21 | PNG72 Papua New Guinea | | Papua New Guinea |
| 22 | PVRVL1997 Papua New Guinea | | Papua New Guinea |
| 23 | XUC014 Papua New Guinea | | Papua New Guinea |
| 24 | PvP01 07 v2 Papua New Guinea | | Papua New Guinea |
| 25 | PvLZCH1476 Myanmar | | Myanmar |
| 26 | PvLZCH1720 Myanmar | | Myanmar |
| 27 | PvLZCH1599 Myanmar | | Myanmar |
| 28 | PvLZCH1708 Myanmar | | Myanmar |
| 29 | PvLZCH1447 Myanmar | | Myanmar |
| 30 | PvP01 07 v2 North Korea | | North korea |
| 31 | PvP01 07 v2 Panama | | Panama |
| 32 | PvP01 07 v2 Nicaragua | | Nicaragua |
| 33 | DTS0721 Peru | | Peru |
| 34 | DTS0791 Peru | | Peru |
| 35 | Peru08 | | Peru |
| 36 | Peru3133 | | Peru |
| 37 | Peru00692 | | Peru |
| 38 | Peru4023 | | Peru |
| 39 | Peru2025 | | Peru |
| 40 | Peru1008 | | Peru |
| 41 | Peru872 | | Peru |
| 42 | Peru858 | | Peru |
| 43 | PvNB43 China | | China |
| 44 | China NB-15 | | China |
| 45 | PvNB48 China | | China |
| 46 | PvNB45 China | | China |
| 47 | China LZCH-20 | | China |
| 48 | China NB-16 | | China |
| 49 | BrazilI | | Brazil |
| 50 | Brazil32 | | Brazil |
| 51 | Belem | | Brazil |
| 52 | PvP01 07 v2 Brazil | | Brazil |
| 53 | Mexico 21-A | | Mexico |
| 54 | Mexico 760-A | | Mexico |
| 55 | Mexico 165-A | | Mexico |
| 56 | Mexico 1086-A | | Mexico |
| 57 | Mexico 980-A | | Mexico |
| 58 | Mexico 330-A | | Mexico |
| 59 | Mexico 203-04 | | Mexico |
| 60 | Mexico 938-A | | Mexico |
| 61 | Mexico 566-A | | Mexico |
| 62 | Mexico 63-08 | | Mexico |
| 63 | Mexico 161-04 | | Mexico |
| 64 | IndiaVII | | India |
| 65 | IndiaNYC | | India |
